# Acute Pupillary Disorders in Children: A 10-Year Retrospective Study of 101 Patients

**DOI:** 10.3390/children10111739

**Published:** 2023-10-26

**Authors:** Giacomo Garone, Marco Roversi, Mara Pisani, Francesco La Penna, Antonio Musolino, Sebastian Cristaldi, Anna Maria Musolino, Amanda Roberto, Gianni Petrocelli, Antonino Reale, Fabio Midulla, Alberto Villani, Umberto Raucci

**Affiliations:** 1 Neurology, Epilepsy and Movement Disorders Unit, Bambino Gesù Children’s Hospital, IRCCS, 00165 Rome, Italy; giacomo.garone@opbg.net; 2 Department of Neuroscience, Mental Health and Sensory Organs, Faculty of Medicine and Psychology, Sapienza University of Rome, 00189 Rome, Italy; 3 Residency School of Pediatrics, University of Rome Tor Vergata, 00133 Rome, Italyantonio.musolino@opbg.net (A.M.); 4 General Pediatric and Emergency Department, Bambino Gesù Children’s Hospital, IRCCS, 00165 Rome, Italy; mara.pisani@opbg.net (M.P.); francesco.lapenna@opbg.net (F.L.P.); sebastian.cristaldi@opbg.net (S.C.); amcaterina.musolino@opbg.net (A.M.M.); antonino.reale@opbg.net (A.R.); alberto.villani@opbg.net (A.V.); 5 School of Pediatrics, Department Mother-Child and Urologic Science, Sapienza University of Rome, 00161 Rome, Italy; amandaro@tiscali.it; 6 Department of Ophthalmology, Bambino Gesù Children’s Hospital, IRCCS, 00165 Rome, Italy; gianni.petrocelli@opbg.net; 7 Department Mother-Child and Urologic Science, Sapienza University of Rome, 00161 Rome, Italy; fabio.midulla@uniroma1.it; 8 Systems Medicine Department, University of Rome Tor Vergata, 00133 Rome, Italy

**Keywords:** pupillary motility, mydriasis, miosis, children, pediatrics

## Abstract

Background: To date, no study has specifically examined children with acute-onset pupillary motility disorders (APMD). Especially in the Emergency Department (ED), it is crucial to distinguish benign and transient conditions from life-threatening or urgent conditions (UCs). The aim of the study is to describe the clinical characteristics of children with APMD and their association with an increased risk of UCs. Methods: We conducted a pediatric retrospective study of APMD referred to ED over a 10-year period. We described the characteristics in the overall sample and in two subgroups divided according to urgency of the underlying condition. Furthermore, we applied a logistic regression model to identify the variables predictive of LT condition. Results: We analyzed 101 patients. In 59.4%, the APMD was isolated. In patients with extra-ocular involvement, the most frequently associated features were altered consciousness, headache, and vomiting. Exposure to toxic agents was reported in 48.5%. Urgent conditions occurred significantly more frequently in older children, presenting bilateral APMD and/or other ocular or extra-ocular manifestations. Conclusions: Our study shows that UCs most commonly occur in patients presenting with bilateral APMD and other associated features. In unilateral/isolated APMD ophthalmological examination, exclusion of toxic exposure and observation until resolution of symptoms should be recommended.

## 1. Introduction

Pupil examination provides an objective assessment of the visual pathway and the autonomic control of the eye. Pupillary inspection is a valuable part of the general, ophthalmological, and neurological examinations, especially in the emergency setting. Pupillary dysfunction may present alone or can be associated with other neurological signs and symptoms. In the latter scenario, it may represent a valuable finding to localize the injury affecting the nervous system. Ocular motility disorders are a not-so-rare condition among the reasons for access to the Emergency Department (ED), accounting for 0.6 per 1000 visits and representing the second most common acute ocular motility disorder in children [1]. Pupillary disorders can underlie a wide spectrum of different disorders [1], some of which can be life-threatening [2]. Only timely and appropriate treatment can prevent severe morbidity and disability in some patients. Underlying causes include traumatic, infectious, inflammatory, neoplastic, vascular, and toxic disorders [3,4].

Nevertheless, no study investigated the differential diagnosis of pupillary motility disorders in children and no specific algorithm based on clinical practice is available. To date, no study has specifically examined the underlying disorders in children with acute-onset pupillary motility disorders. Especially in the ED scenario, it is critical to reliably distinguish benign and transient conditions from (potentially) life-threatening disorders [3,5].

Therefore, through a retrospective analysis of a large cohort of children who presented to the pediatric emergency department of a tertiary hospital, we describe the epidemiology, clinical features, and underlying causes of acute-onset pupillary motility disorders in children, with the goal of identifying clinical features associated with an increased risk of significant underlying neurological abnormalities and improving their recognition in the emergency setting.

## 2. Materials and Methods

A retrospective single-center cohort study was conducted in the ED of the tertiary-care Children’s Hospital, IRCCS, in collaboration with the postgraduate School of Pediatrics, Faculty of Medicine and Surgery. Patients were identified by keyword searching the hospital’s electronic databases and included all patients younger than 18 years and older than 28 days who accessed the emergency department from 1 January 2010 to 31 December 2019, with acute-onset pupillary motility disorders.

The medical records were selected by searching for the keywords “mydriasis”, “miosis”, and “anisocoria” in the fields “history”, “clinical examination”, “diagnosis”. Then, potential cases were manually selected by medical record review. We included patients who presented with a history of unilateral or bilateral pupillary dilation or restriction lasting less than 30 days, who received a diagnosis of mydriasis, miosis, and/or not otherwise specified anisocoria. Both patients complaining of pupillary disorders and patients admitted for other symptoms (e.g., altered consciousness, headache, or visual disturbances) in whom pupillary abnormalities were detected during the clinical examination were included. In the latter case, the pupillary abnormality was considered to be of new onset if it was reasonably related to the same pathology that caused the acutely presenting symptoms; it had never been detected before or noted in the medical record; and it was not explained by any of the known pre-existing medical problems. Exclusion criteria were (1) age less than 29 days and greater than 18 years; (2) previous diagnosis of neurological or ocular pathology with known pupillary motility disorder; and (3) mydriasis due to known instillation of local mydriatics. The following data were extracted from each medical record: age; sex; triage access code; intoxication (if any), either accidental or voluntary, by contact or ingestion, due to poisonous plants, chemical agents, or drugs; type of pupillary disorder, either isolated or combined with other ocular or nonocular signs/symptoms; comorbidities; hospitalization and length of stay (if applicable); medical imaging; specialist advise; and final diagnosis at discharge.

Consultation priority in our ED was based on a 4-color triage coding scale according to the Italian Health System Guidelines in effect during the study period [6]. For the purposes of this study, ED consultation priority was classified as follows:

1) High/intermediate priority: includes patients classified as “code red” (critical medical status) and “code yellow” (severe status, risk of evolution to critical condition);
2) Low/nonurgent priority: includes patients classified as “code green” (fair status, stable vital signs) or “code white” (good status, nonurgent consultation).

The causes of pupillary motility disorders were classified according to the discharge diagnosis made at the end of the diagnostic work-up. Conditions reflecting significant ocular or neurological abnormalities requiring further investigation and intervention (i.e., malformative, neoplastic, cerebrovascular, infectious, demyelinating, degenerative disorders) were classified as urgent conditions (UCs).

Clinical and demographic characteristics were described in the overall cohort and in the two subgroups (patients with and without UC). Each variable was compared between the two subgroups to identify significant differences. After reviewing for appropriateness, Student’s *t*-test and χ2-test were used for statistical comparison of continuous and categorical variables, respectively. In the latter case, the Fisher exact test was used when the expected cell count was less than 5. A logistic regression analysis model was applied to identify clinical characteristics predictive of an underlying UC. Inclusion of variables in the model was based on clinical plausibility and significant differences in χ2 and *t*-tests between UC and non-UC patients. Adjusted odds ratios (OR) and 95% confidence intervals (95%CI) were used as effect measures. Statistical significance was set at two-sided *p* < 0.05 for all tests. Finally, we calculated the negative predictive values of different combinations of signs and symptoms associated with a UC according to the results of the previous analyses. SPSS^®^ 23.0 software platform (IBM^®^, Armonk, NY, USA) was used to perform all statistical analyses.

This study was approved by the ethics committee of our hospital, protocol 2274_OPBG_2020 on 11 November 2020.

## 3. Results

**Figure 1:**
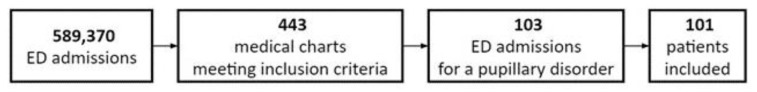
Study flowchart.

During the 10-year study period, a total of 589,370 ED admissions were recorded. After a keyword search, 443 medical charts were manually reviewed and 103 ED admissions from 101 patients were found to meet inclusion criteria (Figure 1), with an observed incidence of 1.75 cases per 10,000 admissions. One patient accessed the ED three times for recurrent episodes of pupillary motility dysfunction.

The clinical and demographic characteristics of the study sample are outlined in Table 1.

The mean age of the patients was 5.0 (SD ± 4.5) years. Most patients were male (60.2%), with a female-to-male ratio of 1:1.5. Priority of consultation was considered high or intermediate in 64 patients (62.1%). In 63 patients (61.2%), pupillary motility disorder was the complaining symptom, while, in the remainder, it was detected during the clinical examination in patients attending the ED for other disorders. The median time from symptom onset to ED admission was 1 day (range 0.13–30 days). In seven patients (6.8%), a history of similar episodes in the past was reported. Exposure to potentially toxic agents with effects on pupillary motility was reported in 48.5% of cases. The intoxication was accidental (84.0%) and by local contact (76.0%) in most cases. Potentially toxic substances included drugs (72.0%), poisonous plants (18.0%), and chemical agents (10.0%). Pupillary disturbance was unilateral in most cases (80.2%) and was classified as mydriasis in 94 cases (93.1%), miosis in 5 (5.0%), or unspecified anisocoria in 2 (1.9%). In 60 cases (59.4%), the pupillary disorder was isolated, in the absence of other ocular or nonocular signs or symptoms. In the remainder, the pupillary disorder was associated with other ocular (15.5%, *n* = 16) and nonocular neurological (34.0%, *n* = 35) signs or both (40.6%, *n* = 41). The most frequently associated signs and symptoms were altered consciousness (19.4%, *n* = 20), headache (15.5%, *n* = 16), vomiting (10.7%, *n* = 11), vision loss (7.8%, *n* = 8), strabismus (4.9%, *n* = 5), and fever (4.9%, *n* = 5). Comorbidities were present in 36 patients (35.0%). Hospitalization was required in 52 cases (50.5%), with a median length of stay of approximately 4 days (range 0–72 days). Neuroimaging was performed in 52 cases (50.5%). Specialist consultation was required in the majority of cases (82.5%), mainly by the ophthalmologist (56.3%) or neurologist (49.5%). Most patients (61.4%, *n* = 62) were discharged with a non-UC, namely a local intoxication (37.6%). The final diagnosis was transient pupillary disorder of unknown cause in 24 patients (23.8%). Because of the transitory nature of the condition, those patients were classified as having a non-UC. Among the UCs (38.6%, *n* = 39), systemic intoxication was found in 10 patients (9.9%), a CNS tumor was detected in 7 (6.9%), a CNS infection was diagnosed in 5 (4.9%), and an ocular disease in 5 (4.9%). Other diagnoses included cerebrovascular disease, encephalopathy, malfunction of a ventriculo-peritoneal shunt, cranial neuropathy, and optic neuritis (see Table 1).

The comparison between patients with or without a UCs is shown in Table 2. 

When comparing patients according to the diagnosis of a UC, we found that those with a UC were significantly older (median age 6.6 vs. 4.0 years, *p* = 0.011), admitted with higher priority (76.9% vs. 54.8%, *p* = 0.025), and mostly entered the ED for a reason other than the pupillary disorder (74.4% vs. 14.5%, *p* < 0.001). Accordingly, most intoxicated patients had a non-UC (62.9% vs. 28.2%, *p* < 0.001). Patients with a UC diagnosed presented significantly more frequently with a bilateral pupillary disorder (46.2% vs. 3.2%, *p* < 0.001) and with other ocular (35.9% vs. 3.2%, *p* < 0.001) or nonocular (71.8% vs. 11.3%, *p* < 0.001) signs and symptoms, mainly impaired consciousness (48.7% vs. 1.6%, *p* < 0.001), headache (25.6% vs. 9.7%, *p* = 0.032), and vomiting (25.6% vs. 1.6%, *p* < 0.001). Accordingly, an isolated pupillary disorder was found in a minority of patients with an underlying UC (15.4% vs. 87.1%, *p* < 0.001).

The logistic regression analysis (dependent variable: UC) is shown in Table 3.

In a logistic regression model that adopted the presence of a UC as the dependent variable, we found that bilateral pupillary disorder (aOR 31.227, *p* = 0.003), other ocular signs (aOR 26.603, *p* = 0.002), and vomiting (aOR 28.604, *p* = 0.034) were the only variables strongly associated with a UC, after adjustment for the other clinically and statistically significant variables in the logistic analysis. In addition, impaired consciousness, although not reaching statistical significance after adjustment for the other variables, was associated with an elevated aOR (11.602). 

The negative predictive value for an isolated, unilateral pupillary disorder was found to be of 93.2%. There were four UC patients without these symptoms and they presented with operated glaucoma, congenital cataract, anisocoria regressed after removal of a foreign body, and transient anisocoria due to ingestion of an Ayurvedic laxative. The first three conditions were classified as urgent because of the risk of ocular compromise, the second because of the risk of systemic intoxication.

## 4. Discussion

To the best of our knowledge, the present study represents the first, large pediatric case series on acute pupillary abnormalities in a pediatric ED. Pupillary disorders occurred in less than 2 cases every 10,000 ED consultations, representing a rare condition in the pediatric emergency setting. Nonetheless, pupillary disorders are of concern to family members or the pediatrician, as the symptom may underlie a potentially severe cause.

The present study shows that the female-to-male ratio is in favor of the latter (F/M ratio: 1:1.5), although this does not represent a statistically significant value, and that the median age of 5.0 years indicates that the condition also affects school and preschool children. Autonomous access or referral to the ED mostly occurred within 48 h from symptom onset, suggesting that pupillary abnormalities are perceived as an alarming sign by caregivers and pediatricians working in outpatient facilities. 

Our data suggest that the underlying conditions are different in children than in adults, where the causes are mainly uveitis, stroke, subarachnoid hemorrhage, acute angle closure glaucoma, major trauma, and demyelinating diseases [2,7,8]. In the pediatric age group, most patients have benign transient causes, especially local exposure to substances inducing transient pupillary dysmotility. In fact, local toxic exposure emerges as the most common condition leading to ED admission for pupillary disorder, accounting for 28.1% of the entire sample examined. From a complete pharmacological history, it was possible to diagnose from the outset that 22 patients had transient “benign” anisocoria related to direct eye contact of ipratropium bromide administered by aerosol, thus avoiding further investigations. In these subjects, the mydriasis was clearly isolated and the patients had normal mental status and no other neurological sign. Ipratropium bromide is a nebulized drug commonly used for obstructive pulmonary diseases, with anticholinergic properties. It may cause complete mydriasis after direct application to the eyes [9,10], and improper placement of the nebulizer mask during aerosol therapy may lead to direct eye contact and cholinergic blockade with subsequent mydriasis [11]. Mydriasis is usually unilateral and transient, resolving in most cases within 24 (or rarely 48) hours. 

More rarely, pupillary abnormalities were induced by local exposure to plants, such as Belladonna (Atropa Belladonna) or thornapples (Datura stramonium) [12,13,14,15,16]. In our cohort, pupillary abnormalities were due to direct contact to Datura stramonium in six patients, Atropa belladonna in two, and by an unidentified plant in another patient. All these plants contain tropane alkaloids such as scopolamine and atropine, with anticholinergic activity. Differently from systemic exposure to tropane alkaloids (which may cause severe anticholinergic intoxication with encephalopathy, flushing, tachycardia, anhidrotic hyperthermia, nonreactive mydriasis, urinary retention, and encephalopathy with delirium and hallucinations), local exposure to these agents is usually milder [17]. 

In our cohort, only one out of nine patients presenting with anticholinergic toxicity due to contact exposure to poisoning plants reported systemic signs (transient dizziness).

Sometimes, identification of the responsible substance is not obvious, as the exposure to the toxic agents is not always noticed or referred at the time of ED consultation. Of note, in our cohort, about a quarter of ED admissions for pupillary abnormalities have been classified as transient unexplained disorders. It is possible that a portion of these cases could be caused by unidentified exposure to toxic substances. In cases of unilateral unexplained mydriasis, a pilocarpine test can be useful in distinguishing the effect of an anticholinergic substance from a central or peripheral nervous system injury. In fact, through its direct parasympathomimetic action, instillation of an eye drop of 1% pilocarpine causes constriction of the pupil by stimulating the sphincter pupillae. In the presence of a mydriatic agent (or direct damage to the pupillary sphincter due to trauma or surgery), the affected eye will be unresponsive to pilocarpine instillation, while, in the case of third nerve palsy or midbrain lesion, it will constrict. In all cases of unexplained, likely pharmacologically induced anisocoria, further toxicological investigations should be performed in order to exclude exposure to potentially harmful substances or drug abuse [18,19]. 

In our study, systemic intoxications represented the first cause of UCs. They were classified as UCs regardless of the clinical status, considering the potential for rapid clinical deterioration in many toxidromes imposes, to reliably identify the offending substance, to monitor for evolving disturbances, and to perform specific treatments where necessary. Causative agents included drugs (benzodiazepines, valproic acid, scopolamine, antidepressants, and antihistaminergic agents) and substances of abuse (ethanol and cannabinoids). Systemic intoxications usually cause bilateral pupillary disturbances, and neurologic or systemic signs dominate the clinical presentation. Nevertheless, the pupillary disorder may constitute a helpful element to identify the offending agent [17].

Other UCs in our cohort encompass a broad range of individually rare ocular and neurological disorders, including CNS malignancies and infections, congenital cataracts, cerebrovascular diseases, VPS malfunctioning, optic neuritis, and other cranial neuropathies. In most of these cases, pupillary disorders were associated with abnormal systemic, neurological, or ophthalmological findings. 

According to our analysis, the occurrence of bilateral pupillary disorders, the coexistence of other ocular disorders or vomiting were strong predictors of an underlying UC. In addition, impaired consciousness was associated with an 11-fold increase in the odds of an underlying UC, although not reaching statistical significance, probably because of the relative low frequency in the overall cohort.

These findings probably reflect the higher incidence of these features in patients with systemic intoxications or other encephalopathy due to widespread CNS injury and conditions causing raised intracranial pressure.

To these findings, other obvious indications to further investigations—that were not included in the logistic regression model because of their low occurrence in our cohort—should be added, such as papilledema, ataxia, seizures, and neck stiffness.

In contrast, the presentation of unilateral, isolated (namely, not associated with other ocular, neurological, or systemic signs or symptoms) pupillary disorder was found to be associated with a negative predictive value for a UC of 93.2%. The four false positive cases (namely, patients with unilateral, isolated disorder but with an underlying UC) were represented by three ocular conditions of surgical interest and one potential systemic intoxication. Given these data, it seems reasonable to always recommend ophthalmological examination (to exclude ocular diseases), pilocarpine test (where appropriate), and toxicological investigations (if pharmacologically induced anisocoria is suspected and the offending drug cannot be easily identified). Once an ocular disorder and a serious intoxication are excluded, observation until resolution of symptoms should be performed.

Mostly because of its retrospective design, this study suffers from some limitations that may affect our conclusions. First, ED assessment of pupillary disorders (as extracted from medical records) may not ensure an accurate clinical evaluation of rarer and difficult-to-assess ocular disorder, although many patients received neurologist or ophthalmologist consultations. However, it is unlikely that pupillary disorders associated with serious conditions (i.e., the purpose of this study) have been missed. Moreover, several patients (especially non-hospitalized patients) did not receive a definite diagnosis for the acute pupillary disorder, reflecting the only partial knowledge of conditions causing acute pupillary disorders in this age group, especially in patients with transient, isolated, and not recurrent manifestations that did not undergo diagnostic investigations. In addition, no clinical protocol for assessment was applied during the study period, making management of our cohort highly heterogenous. Albeit these limitations largely affect our ability to draw firm conclusions on the exact etiology and management of non-LT pupillary disturbances in children, they are unlikely to affect the validity of our conclusion about the identification of LT conditions. Finally, our logistic regression model helps in identifying clinical features associated with a greater probability of potentially threatening conditions, but they do not orient towards a specific underlying disorder nor guide the choice of the most indicated investigations.

## 5. Conclusions

Although rare, pupillary disturbances are a challenging dilemma in the acute setting. Our study shows that UCs account for a significant proportion of acute pupillary disorders but most commonly occur in patients presenting for other medical complaints, with bilateral disorder and associated signs and symptoms (especially occurrence of other ocular deficits, vomiting, altered mental status, and other focal neurological signs). 

In cases of unilateral, isolated pupillary disturbances, ophthalmological examination, exclusion of toxic exposure, and observation until resolution of symptoms appears as a viable and efficacious way of managing these troublesome young patients.

Our data suggest that a standardized approach to acute pupillary disturbances may ensure a reliable diagnosis and restrict invasive or expensive investigations to patients with high clinical suspicion of an underlying harmful condition. 

## Figures and Tables

**Table 1:** Characteristics of study sample.

Total Number—No.	103 ^a,b^
Age—mean ± SD (range)	5.0 ± 4.5 (0.07–15.96)
Males—no. (%)	62 (60.2)
Female-to-male ratio	1:1.5
Triage	
High/intermediate priority	64 (62.1)
Low priority	39 (37.9)
Reason for entering the ED—no. (%)	
Pupillary disorder	63 (61.2)
Other	38 (37.6)
Time from onset (days)—median ± IQR (range)	1 ± 2 (0.13–90)
History of similar episode—no. (%)	**7** (6.8)
Intoxication—no. (%)	50 (48.5)
Accidental	42 (84.0)
Voluntary	8 (16.0)
Exposure to toxic substance—no. (%)	
Contact	38 (76.0)
Ingestion	12 (24.0)
Type of toxic substance	
Poisonous plants	9 (18.0)
Chemical agents	5 (10.0)
Drugs	37 (72.0)
Pupillary disorder—no. (%)	
Mydriasis	94 (93.1)
Myosis	5 (5.0)
Unspecified anisocoria	2 (1.9)
Unilateral	81 (80.2)
Bilateral	20 (19.8)
Isolated pupillary disorder—no. (%)	60 (59.4)
Other ocular signs/symptoms—no. (%)	16 (15.5)
Loss of vision	8 (7.8)
Strabismus	5 (4.9)
Ptosis	3 (2.9)
Papillary edema	3 (2.9)
Nonocular signs/symptoms—no. (%)	35 (34.0)
Impaired consciousness	20 (19.4)
Headache	16 (15.5)
Vomiting	11 (10.7)
Fever	5 (4.9)
Focal deficits	3 (2.9)
Ataxia	3 (2.9)
Seizure	2 (1.9)
Neck stiffness	2 (1.9)
Hypotonia	2 (1.9)
Vertigo	1 (0.9)
Comorbidities—no. (%)	36 (35.0)
Hospitalization—no. (%)	52 (50.5)
Length of stay (days)—median ± IQR (range)	4 ± 13 (0–72)
Discharge—no. (%)	51 (49.5)
Medical imaging—no. (%)	52 (50.5)
Head CT scan only	31 (30.1)
Head MRI only	4 (3.9)
Both head CT and MRI	13 (10.0)
VEP/ERG	9 (8.7)
Specialist advice—no. (%)	85 (82.5)
Ophthalmologist	58 (56.3)
Neurologist	51 (49.5)
Intensive care physician	19 (18.4)
Neurosurgeon	10 (9.7)
Neuropsychiatrist	4 (3.9)
Diagnosis	
Urgent condition—no. (%)	39 (38.6)
Systemic intoxication	10 (9.9)
CNS tumor	7 (6.9)
CNS infection	5 (4.9)
Ocular disease	5 (4.9)
Cerebrovascular disease	3 (2.9)
Other encephalopathy *	3 (2.9)
Malfunction of VPS	2 (1.9)
Cranial neuropathy	2 (1.9)
Optical neuritis	2 (1.9)
Nonurgent condition—no. (%)	62 (61.4)
Local intoxication	38 (37.6)
No diagnosis	24 (23.8)

^a^ Percentages were calculated accounting for missing values. ^b^ Calculations were made accounting for one patient admitted three times to the ED. * Coma in caustic ingestion, coma from diabetic ketoacidosis, and transient undetermined coma.

**Table 2:** Comparison of patients with or without an urgent condition (UC) ^a^.

	UC	Non UC	*p*-Value
Total number—no.	39	62	
Age—mean ± SD (range)	6.6 ± 5.3 (0.1–16.0)	4.0 ± 3.7 (0.1–14.3)	**0.011**
Males—no. (%)	20 (51.3)	40 (64.5)	0.216
Triage			
High/intermediate priority	30 (76.9)	34 (54.8)	**0.025**
Low priority	9 (23.1)	28 (45.2)	**0.025**
Reason for entering the ED—no. (%)			
Pupillary disorder	10 (25.6)	53 (85.5)	**<0.001**
Other	29 (74.4)	9 (14.5)	**<0.001**
Time from onset (days)—median ± IQR (range)	1 ± 2 (1–30)	1 ± 0 (0.13–90)	**0.013**
History of similar episode—no. (%)	4 (57.1)	3 (42.9)	0.257
Intoxication—no. (%)	11 (28.2)	39 (62.9)	**0.001**
Accidental	5 (45.5)	37 (94.9)	**0.001**
Voluntary	6 (94.9)	2 (5.1)	**0.001**
Exposure to toxic substance—no. (%)			
Contact	0 (0)	38 (97.4)	**<0.001**
Ingestion	11 (100)	1 (2.6)	**<0.001**
Type of toxic substance—no. (%)			
Poisonous plants	0 (0)	9 (23.1)	0.177
Chemical agents	5 (45.5)	0 (0)	**<0.001**
Drugs	7 (63.6)	30 (76.9)	0.445
Pupillary disorder—no. (%)			
Mydriasis	36 (92.3)	58 (93.5)	1.000
Myosis	3 (7.7)	2 (3.2)	0.372
Unilateral	21 (53.8)	60 (96.8)	**<0.001**
Bilateral	18 (46.2)	2 (3.2)	**<0.001**
Unspecified	0 (0)	2 (3.2)	0.521
Isolated pupillary disorder—no. (%)	6 (15.4)	54 (87.1)	**<0.001**
Other ocular signs/symptoms—no. (%)	14 (35.9)	2 (3.2)	**<0.001**
Loss of vision	6 (15.4)	2 (3.2)	0.052
Strabismus	5 (12.8)	0 (0)	0.007
Ptosis	3 (7.7)	0 (0)	0.055
Papillary edema	3 (7.7)	0 (0)	0.055
Nonocular signs/symptoms—no. (%)	28 (71.8)	7 (11.3)	**<0.001**
Impaired consciousness	19 (48.7)	1 (1.6)	**<0.001**
Headache	10 (25.6)	6 (9.7)	**0.032**
Vomiting	10 (25.6)	1 (1.6)	**<0.001**
Fever	4 (10.3)	1 (1.6)	0.072
Focal deficits	3 (7.7)	0 (0)	0.055
Ataxia	3 (7.7)	0 (0)	0.055
Seizure	2 (5.1)	0 (0)	0.147
Neck stiffness	2 (5.1)	0 (0)	0.147
Hypotonia	2 (5.1)	0 (0)	0.147
Dizziness	0 (0)	1 (1.6)	1.000
Comorbidities—no. (%)	12 (30.8)	24 (38.7)	0.497
Hospitalization—no. (%)	36 (92.3)	16 (25.8)	<0.001
Length of stay (days)—median ± IQR (range)	9 ± 17 (0–72)	1 ± 1 (0–11)	
Discharged (no hospitalization)—no. (%)	3 (7.7)	46 (74.2)	<0.001
Medical imaging—no. (%)	28 (71.8)	24 (38.7)	0.001
Head CT scan only	13 (33.3)	18 (29.0)	0.648
Head MRI only	1 (2.6)	3 (4.8)	1.000
Both head CT and MRI	12 (30.8)	1 (1.6)	<0.001
VEP/ERG	7 (17.9)	2 (3.2)	0.026

^a^ Percentages were calculated accounting for missing values.

**Table 3:** Logistic regression analysis (dependent variable: life-threatening condition).

Variables	aOR	C.I. 95%	*p*-Value
Age (years)	1.014	0.842–1.221	0.883
Time from onset (days)	1.000	0.939–1.065	0.997
Reason for entering the ED (other)	1.946	0.311–12.164	0.477
Intoxication (yes)	0.321	0.057–1.814	0.199
Pupillary disorder (bilateral)	31.227	3.327–293.056	**0.003**
Other ocular signs/symptoms (yes)	26.603	3.206–220.741	**0.002**
Impaired consciousness (yes)	11.602	0.807–166.693	0.071
Headache (yes)	0.131	0.011–1.620	0.113
Vomiting (yes)	28.064	1.277–616.633	**0.034**

## Data Availability

The datasets generated and analyzed during the current study are not publicly available due to privacy protection but are available from the corresponding author on reasonable request.

## Abbreviations

APMD: acute-onset pupillary motility disorders, CI: confidence interval, ED: Emergency Department, LT: life-threatening, aOR: adjusted odds ratio, UC: urgent condition.
